# Highlighting the impact of aging on type I collagen: label-free investigation using confocal reflectance microscopy and diffuse reflectance spectroscopy in 3D matrix model

**DOI:** 10.18632/oncotarget.7385

**Published:** 2016-02-14

**Authors:** Marie Guilbert, Blandine Roig, Christine Terryn, Roselyne Garnotel, Pierre Jeannesson, Ganesh D. Sockalingum, Michel Manfait, François Perraut, Jean-Marc Dinten, Anne Koenig, Olivier Piot

**Affiliations:** ^1^ MéDIAN-Biophotonique et Technologies pour la Santé, Université de Reims Champagne-Ardenne, CNRS UMR 7369 MEDyC, UFR de Pharmacie, SFR CAP Santé, Reims, France; ^2^ CEA, LETI, Minatec campus, Grenoble, France; ^3^ Plate-forme Imagerie Cellulaire et Tissulaire, Université de Reims Champagne-Ardenne, Reims, France

**Keywords:** Type I collagen aging, 3D matrix, confocal reflectance microscopy, diffuse reflectance spectroscopy, ATR infrared imaging, Gerotarget

## Abstract

During aging, alterations of extracellular matrix proteins contribute to various pathological phenotypes. Among these alterations, type I collagen cross-linking and associated glycation products accumulation over time detrimentally affects its physico-chemical properties, leading to alterations of tissue biomechanical stability. Here, different-age collagen 3D matrices using non-destructive and label-free biophotonic techniques were analysed to highlight the impact of collagen I aging on 3D constructs, at macroscopic and microscopic levels. Matrices were prepared with collagens extracted from tail tendons of rats (newborns, young and old adults) to be within the physiological aging process. The data of diffuse reflectance spectroscopy reveal that aging leads to an inhibition of fibril assembly and a resulting decrease of gel density. Investigations by confocal reflectance microscopy highlight poor-fibrillar structures in oldest collagen networks most likely related to the glycation products accumulation. Complementarily, an infrared analysis brings out marked spectral variations in the Amide I profile, specific of the peptidic bond conformation and for carbohydrates vibrations as function of collagen-age. Interestingly, we also highlight an unexpected behavior for newborn collagen, exhibiting poorly-organized networks and microscopic features close to the oldest collagen. These results demonstrate that changes in collagen optical properties are relevant for investigating the incidence of aging in 3D matrix models.

## INTRODUCTION

The aging process constitutes a field of major interest in our society since it represents a progressive, irreversible, and multidimensional mechanism affecting all people. Biological aging starts before the visible clinical signs, in particular by metabolism variations affecting long-lived molecules such as deoxyribonucleic acid (DNA) or structural proteins. If age-related DNA damages and resulting-cellular senescence represent a key concept of aging strongly investigated [1, 2], age-related modifications of matrix proteins play a crucial role in chronological aging and associated-physiopathology. Indeed, modifications of extracellular matrix (ECM) components affect tissue biomechanical properties *in vivo*, leading to a decline of both tissue integrity and function [3, 4]. Among these matrix compounds, type I collagen represents the most abundant long-life protein in humans, and the key fibrillar structural component of several tissues such as skin, bone, or tendon [5]. Its structure consists of three parallel polypeptide strands (two-α_1_ and one-α_2_ chains) coiled together into a right-handed triple helix; triple helices then assemble into supramolecular structures corresponding to collagen fibrils and fibers [6, 7]. This scaffold is fundamental for tissue integrity in governing their biomechanical properties, thermal stability, mechanical strength, and interactions with other biomolecules and cells [8, 9]. Networks of type I collagen can be obtained *in vitro*, and 3D matrices have been largely developed as collagen tissue substitutes in cell biology or tissue engineering [10, 11].

Due to its long biological lifespan, type I collagen undergoes several modifications of its physico-chemical properties over time, leading to tissue dysfunction in the elderly [12]. These age-related changes are mainly due to the non-enzymatic glycation process, the major post-translational alteration of long-lived proteins described *in vivo* [13, 14]. This mechanism, well-known as the Maillard reaction, consists in the spontaneous reaction between oxo-group of circulating reducing sugars (mostly glucose), and reactive amino-acid groups of the collagen side chains (essentially lysine residues), resulting in the irreversible formation of the so-called advanced glycation end products (AGEs) [15, 16]. These late glycation products, most of which exhibit fluorescent properties, are implicated in aging and its associated-complications [17]. As a result of their cross-linking effect, AGEs have a direct impact on the triple helix properties which becomes less flexible, less soluble, and more resistant to enzyme digestion [18]. Such alterations affect the ECM structural scaffold, leading to changes in biomechanical properties, in particular an increasing stiffness, and loss of functional integrity of collagenous tissues [3, 19]. The evaluation of age impact on type I collagen properties is commonly assessed *in vitro* by quantification of cross-link due to AGEs using mass spectroscopic or chromatographic assays [4]. However, these techniques require a deep enzymatic fragmentation into small peptides and so cannot be used directly on *ex vivo* collagen 3D constructs or *in vivo* tissues. Moreover, they do not provide information about the age effect at the supramolecular level such as fiber assembly and network organization of type I collagen. Age incidence on such structural parameters can be evaluated *in vitro* through investigation of polymerization efficiency and structural properties of type I collagen in 3D experimental model [20, 21].

In the present study, we investigated the influence of aging in collagen 3D matrix model, using a combination of label-free biophotonic tools allowing to access complementary data. For that, confocal reflectance microscopy (CRM) was applied to image the different-age collagen 3D constructs, providing information on the microscopic organization of networks. CRM allows a direct monitoring of fibril and matrix structure in 3D, using intrinsic optical properties of collagen based on the light back-scattering [22]. Complementarily, the 3D matrices from different-age collagens were analysed by diffuse reflectance spectroscopy (DRS) which can be used on biological samples to determine their specific optical properties [23, 24]. The scattering spectra specific of type I collagen may reflect structural differences occurring with collagen age. In parallel, the *in vitro* gelation process was monitored in real-time to evaluate the age incidence on the collagen ability to assemble into fibrillar structures. The study was completed with a spectral analysis of 3D different-age collagen matrices using Fourier-transform infrared (FTIR) microspectroscopy in attenuated total reflection (ATR) mode; infrared spectroscopy is a vibrational technique highly sensitive to both molecular and conformational protein changes, that permits to probe age-related modifications of collagen [25, 26]. This work, based on a multimodal biophotonic approach, aims at highlighting, at both macroscopic and microscopic levels, the incidence of collagen aging directly on 3D constructs, in a label-free and non-destructive way.

## RESULTS

### Biochemical properties of type I collagen as a function of age

**Figure 1:**
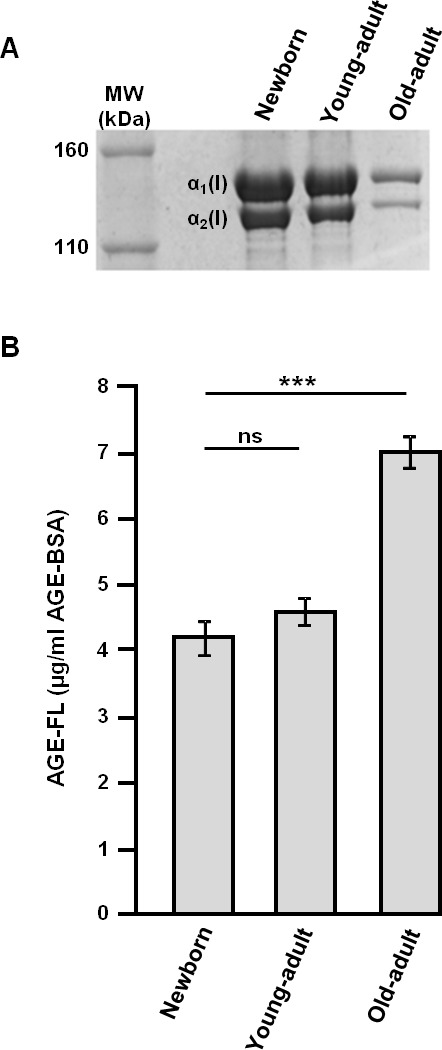
Biochemical characterization of different-age type I collagens Electrophoretic profile **A.** and fluorescent-AGEs quantification **B.** of different-age type I collagens extracted from newborn, young-adult, and old-adult rats. Data are representative of three-independent experiments (mean ± SD). ***p < 0.001 significant difference; ns, not significant.

The electrophoretic profile was studied for verifying the purity of extracted different age type I collagens, and the AGEs-related fluorescence intensity was measured for evaluating the AGEs accumulation in collagen with aging [21, 25]. As shown in Figure 1A, two bands migrating between 160 and 110 kDa are detected in all different-age collagens, corresponding to the α_1_(I) and α_2_(I) chains characteristic to native type I collagen. For the old-adult collagen specimen, bands exhibit a lower density and a delayed migration, indicating a decrease of both collagen mobility and solubility with increasing age. The quantification of fluorescent-AGEs (Figure 1B) shows a significant increase of their content for the old-adult collagen (increase of about 40% compared to the young-adult collagen); this accumulation of AGEs, resulting from the non-enzymatic glycation reaction occurring with chronological aging, is directly linked to the observed changes in collagen electrophoretic properties.

### Impact of collagen-age on gel formation

**Figure 2:**
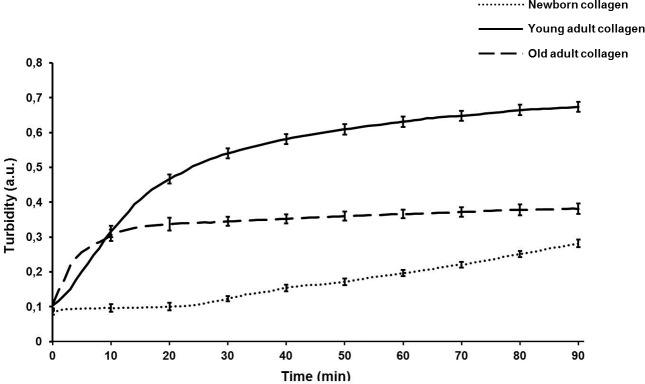
Kinetics profile of *in vitro* fibrillogenesis of different-age type I collagens Gel formation of different-age type I collagens was monitored by measuring the absorbance at 400 nm every minute during 90 min after neutralization of the pH collagen solution. Each measure point is the mean of three-independent experiments (mean ± SD).

The ability of different-age collagens to assemble into a gel was evaluated using a spectrophotometer recording the absorbance at 400 nm for 90 min under physiological pH and temperature, and raw absorbance data were also converted into turbidity [27]. The results of this kinetic study (Figure 2) are displayed as typical sigmoid curves. The young-adult collagen (dark line) exhibits a kinetic curve with three phases: a short lag phase (0-3 min), an exponential phase (3-20 min) corresponding to the gel formation due to fibrils assembly, and a plateau phase (after 30 min) characterizing the collagen gel density and its stability. The old-adult collagen (dashed line) shows a faster gelation process: the exponential phase starts at the initial time, without lag phase, and the plateau is reached after only 10 min. Furthermore, the maximal turbidity achieved for the oldest collagen is considerably lower compared to the young-adult one (0.38 *versus* 0.67 respectively), indicating a critical decrease of gel density. The kinetic curve of the collagen extracted from newborn rats (*dotted line*) presents an atypical profile: a very slow gel formation without reaching a plateau after the 90 min of monitoring, suggesting gel instability with a poor density. This *in vitro* fibrillogenesis assay demonstrates that collagen age has an important impact on the fibril assembly and resulting gel density. The newborn collagen is not able to organize it into a strengthened and tensile 3D system. Conversely, the gel formation for the oldest collagen is faster, but the decrease of gel density indicates an inhibition of fibril assembly with aging.

### Macroscopic level analysis of different-age collagen 3D matrices by DRS

**Figure 3:**
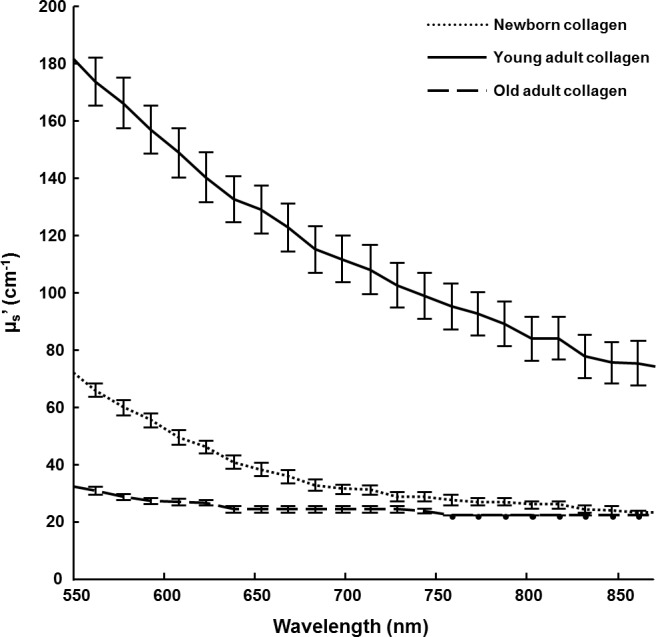
DRS analysis of 3D matrices as a function of collagen age The scattering coefficient (μ_s_’) of 3D matrices from different-age type I collagens was calculated for wavelengths ranging from 550 to 900 nm.

DRS measurements provide some information on the scattering properties of collagen, and could help to compare samples as a function of collagen aging. The Figure 3 displays the reduced scattering coefficient (μ_s_’) calculated for each age condition. The curves shapes are significantly different between the three different-age collagens. This spectral distinction highlights differences of structural properties in the collagen fiber network with age since DRS technique is sensitive to both fiber organization and dimensions. The young-adult collagen presents the highest scattering level compared to the two other age groups, indicating the best fibrillar organization. The scattering spectra of the oldest collagen are very low, suggesting a loss of collagen assembly with aging. For the newborn collagen, it exhibits an intermediate scattering profile; in this case, it could be suggested that 3D matrices from newborn collagen suffer from a lack of fibrillar organization. Microscopic analyses using CRM were then conducted in order to link these observations with the fiber microscopic behavior of different-age collagens in 3D matrices.

### Microscopic level analysis of different-age collagen 3D matrices by CRM

**Figure 4:**
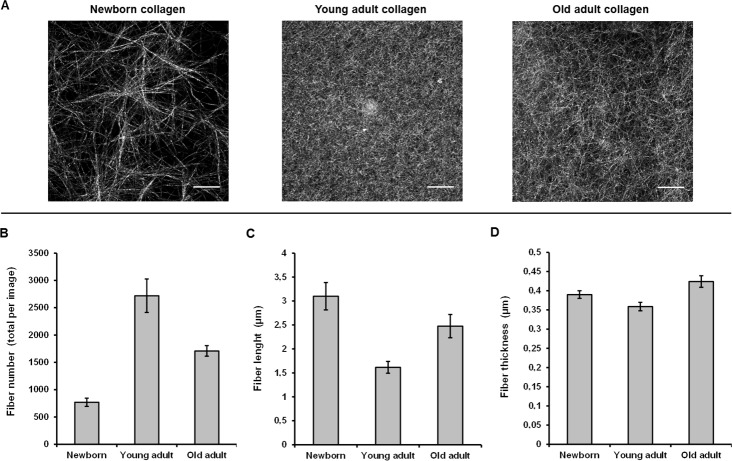
CRM analysis of 3D matrices as a function of collagen age Confocal images **A.** are the Maximum Intensity Projection on a 20 μm stack with z = 0.5 μm. Fiber number **B.**, length **C.**, and thickness **D.** were quantified using ImageJ^®^ software.

Confocal reflectance microscopy allows providing high-resolution images based on the intrinsic optical properties of collagen. The sequential focal planes along the z axis can be reconstructed in order to give a 3D visualization of the network organization as a function of collagen age. Morphological analysis of the confocal images (Figure 4A) indicates that the young-adult collagen is organized in a dense fibrillar network with homogeneous repartition of collagen fibers. The newborn collagen exhibits a network of bundles with important lacks of reflectance signal. Since the confocal reflectance microscopy is sensitive to fiber structure, these data indicate that collagen from newborn presents important defects of fiber formation; the gels being prepared with the same quantity of type I collagen for each age-condition. Concerning the oldest collagen, the network appears heterogeneous and less dense compared to the control one (collagen from young-adult rats). These data correlate with the kinetic fibrillogenesis assay, and suggest that changes in collagen behavior due to age-related cross-linking affect the 3D supraorganization.

Quantitative analysis of CRM data was performed on confocal images. The mean fiber number (Figure 4B), fiber length (Figure 4C), and fiber thickness (Figure 4D) were calculated; results indicate that the young-adult collagen presents the most important number of detected fibers, confirming a higher fibril assembly during gelation. The oldest collagen shows an increase of both fiber length and fiber thickness compared to the young-adult collagen, indicating that post-translational modifications occurring with aging affect the structural stability of collagen fibrils/fibers. The newborn collagen presents a different behavior as expected, with higher fiber length and thickness, and lower fiber number than for the young-adult collagen. These results are coherent with previous data observed on isolated collagen fibrils, using polarized-resolved second harmonic generation microscopy [28].

### ATR-IR analysis of different-age collagen 3D matrices

**Figure 5:**
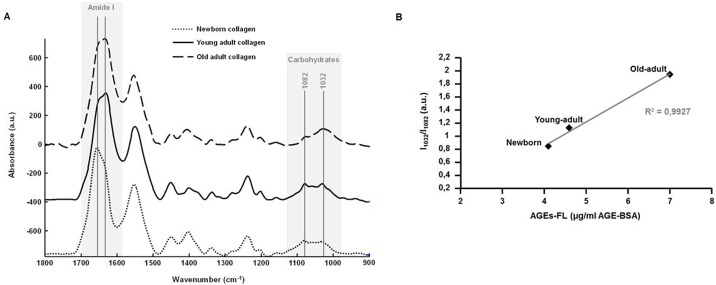
ATR-FTIR analysis of 3D matrices as a function of collagen age Comparison between ATR-FTIR spectra recorded on 3D matrices of different-age type I collagens **A.** with gray zones indicating spectral regions that exhibit spectral variations as a function of collagen age. A correlation analysis was performed between the ratio of infrared intensities of the 1032 and 1082 cm^−1^ peaks (I_1032_/I_1082_), and the mean fluorescence intensity measured for each age specimen **B.**.

Collagen 3D matrices were analysed in infrared microspectroscopy, using the ATR imaging mode. As shown in Figure 5A, ATR-IR spectra allow highlighting spectral differences, especially for the Amide I peak (1660 cm^−1^) and the carbohydrate spectral band (1100-900 cm^−1^) bands. The up-shift in the Amide I peak (from 1656 cm^−1^ for the newborn collagen to 1632 cm^−1^ for the young-adult and old-adult collagens) reflects modifications of collagen conformation in 3D fibrillar network under age. The profile of the carbohydrate band exhibits changes in the ratio between the peak specific to the protein signal (1082 cm^−1^) and the peak specific to the carbohydrate signal (1032 cm^−1^); with collagen aging, the signal of carbohydrates increases to become predominant over the total protein signal, reflecting the accumulation of AGEs. To confirm these observations, the ratio of the intensities of the two 1032 and 1082 cm^−1^ infrared peaks (I_1032_/I_1082_) was computed for each age, and correlated with the AGEs-linked fluorescence intensity (data from Figure 1B). The Figure 5B displays the correlation analysis and the corresponding trendline that gives a correlation coefficient greater than 99% (0.9927), showing a high positive correlation between the infrared features of collagen samples and their fluorescence intensity related to AGEs.

## DISCUSSION AND CONCLUSIONS

In this study, different-age type I collagens were compared in 3D experimental model using label-free and direct tools allowing to obtain information at both macroscopic and microscopic scales. Taken together, our data show the potential of the combination of DRS and CRM methodologies to characterize the changes occurring in 3D matrices as a function of collagen age. Firstly, we demonstrate the correlation between network alterations observed in 3D old-adult collagen matrices and the accumulation of AGEs in collagen due to aging. Secondly, we reveal the non-typical behavior of newborn collagen which is not able to assemble into fibers, and consequently is not able to organize it into a 3D dense network.

Data obtained with DRS measurements are coherent with gelation experiment; collagen aging inhibits the fibril assembly in 3D configuration, resulting in a decrease of gel density and weak scattering properties for the oldest collagen compared to the two other ages. These observations are reinforced with the microscopic measures since the old-adult collagen network is characterized by fewer, longer, and thicker fibrillar structures, compared to its young-adult counterpart. The resulting poor-fibrillary network is directly linked to the accumulation of glycation products under age-affect (as confirmed by fluorescence measurement), which modifies the collagen properties and leads to modifications of fibril arrangement [18, 19]. Purified type I collagen extracted from rat tendons is able to spontaneously assemble into fibrils *in vitro* because collagen molecules are free to bind to other collagen molecules [29]; however, it was previously established that collagen aging leads to a change of the monomer/dimer collagen strands [21]. Over time, the AGEs accumulation is responsible for additional intra- and inter-crosslinks in collagen which result in monomeric changes with aging. Due to such a sterically overcrowded effect, there is a lower effective concentration of available monomers in the oldest collagen to assemble with free counterparts into fibers. These modifications affecting at the monomeric level the 3D assembly of the old-adult collagen [21], can be detected at the collagen fiber scale as well as at the global network level.

Fourier-transform infrared (FTIR) spectroscopy was used here for highlighting the vibrational modes of collagen involved in aging process. Among the different working configurations employed in FTIR analyses, using the attenuated total reflection (ATR) mode provides significant advantages. They are mainly related to the internal reflexion of the infrared light due to the high refractive index of the Germanium crystal, resulting in enhanced spatial resolution, and giving the opportunity to analyze samples with strong absorbing components, such as water [30]. Consequently, this approach is very suitable for investigating age-dependent spectral variations in our 3D hydrated collagen constructs. Mean ATR-FTIR spectra from 3D different-age collagen models show consequent variations for the Amide I band (1660 cm^−1^) and the spectral region specific to carbohydrates (1100-900 cm^−1^). The significant up-shift of the Amide I peak observed in the newborn-collagen case clearly reflects conformational changes in the collagen secondary structure and the resulting 3D network. The intensity ratio between 1032 cm^−1^ (C-OH bonds specific to carbohydrates) and 1660 cm^−1^ (Amide I) vibrations is correlated with the fluorescent-AGEs accumulation over age, indicating that the carbohydrate band is an infrared marker of age-related modifications in collagenic matrices. These results confirm previous works conducted on *in vitro*-glycated collagen samples [25], in particular by Roy *et al*. who used ATR-FTIR to quantify glycation effects in collagen gels after incubation with reducing sugars [31].

In the present study, we also highlight the unexpected behavior of newborn collagen which exhibits an intermediate profile in DRS analysis. A same observation can be made for microscopic investigation using CRM: a parabolic effect is observed when the three different-age groups are compared together. Measurements of fiber number, length, and diameter, tend to indicate that the microscopic structure of the collagen extracted from newborn rats is close to the old-adult collagen. These data are consistent with our previous work on 2D isolated fibrils, reflecting the atypical behavior of newborn collagen [28]. Indeed, it was previously shown at the microscopic scale that the newborn collagen fibrils exhibit a high molecular order, close to the data obtained from the old-adult collagen specimen; this reflects a less complex assembly of individual fibrils compared to the young-adult collagen which presents a low molecular order as a signature of a more complex structural assembly. However, at the 3D supraorganized level, the biomechanical properties (as elasticity) can be ascribed to more macroscopic scale effects related to age incidence, such as the impact of AGEs on the collagen fiber assembly into a 3D network [21], highlighting that the high structural differences microscopically observed between newborn and young-adult collagens are not necessarily correlated with their macroscopic elasticity properties [28]. Moreover, type I collagen extracted from newborn exhibits an important defect of fibrillogenesis and a poor-organized 3D network since it is not able to form a dense gel after the duration of the experiment. In addition, the shift of the Amide I peak observed on spectra from newborn samples indicates changes in protein secondary structure. All these results can be explained by the natural history of collagen from gestation to childhood periods. At birth, collagen fibers present an immaturity along with a high metalloproteinase activity and a defect of fiber alignment despite of a high content in type I collagen [32, 33]. It is during the childhood that collagen acquires its maturity through stabilization by enzyme-derived cross-linking and alignment capacity in parallel with the establishment of mechanical forces in collagenous tissues. Since the determination of optical properties of collagen can be processed to detect the lack of collagen fibrillary assembly, our approach could be very helpful for label-free and non-invasive characterisation of immature or defective network in pathological situations such as osteogenesis imperfecta that results in crucial decrease of rigidity and mechanical stability of collagen molecules [34].

To conclude, this work highlights that scattering properties of collagen (measured by DRS) help to reflect the changes in 3D ultrastructural organization as a function of collagen age. Experiments were performed directly on 3D matrices that provide a physiologic concentration of collagen for cell analysis and engineering of collagenous substitutes [11, 21]. By taking into account age-related modifications in these culture models, our data confirm the suitability of scattering properties of type I collagen as a potential *in vivo* marker of aging or associated-complications such as diabetes or atherosclerosis. Complementarily, the quantitative analysis of CRM images allows obtaining some information on the fibrillar microscopic state. Thus, we demonstrate the feasibility to combine non-invasive biophotonic methodologies for investigating changes in collagen optical properties that reflect age-related modifications of 3D collagen at molecular, microscopic, and macroscopic levels.

## MATERIALS AND METHODS

### Extraction of different-age type I collagens

Type I collagens were extracted from tail tendons of different-age Sprague-Dawley rats, 4 to 6-day old (newborns), 2-month old (young adults), and 2-year old animals (old adults). To ensure a good homogeneity in each age group, tail tendons from rats of the same age were pooled, and type I collagens were prepared as previously described [11]. Briefly, acid-soluble collagen samples were extracted using 0.5 M acetic acid, then purified by dialysis against distilled water, lyophilized, and stored at −80°C until further use. This protocol, without pepsinization, allows obtaining native, purified, and fibrillar type I collagens with intact telopeptides, in contrast with most of those commercially available [35].

### Biochemical characterization of different-age type I collagens

Biochemical properties of different-age extracted collagens were evaluated after solubilization at 2 mg/ml in 0.018 M acetic acid (v/v). Firstly, electrophoretic properties were estimated by 5% sodium dodecyl sulfate-polyacrylamide gel electrophoresis (SDS-PAGE) after denaturation through heating at 90°C for 2 min. Gels were stained with Coomassie Brillant Blue R250 for revealing characteristic bands of type I collagen (α_1_ and α_2_ chains). Secondly, the fluorescence specific to AGEs was measured using a Shimadzu RF-5000 spectrofluorometer (Shimadzu, France) at 380 nm excitation and 440 nm emission. AGE-modified bovine serum albumin (BSA) was used to create a standard curve.

### Preparation of 3D collagen matrices

Different-age type I collagen 3D matrices were prepared by neutralization of collagen solution with NaOH according to the protocol previously developed [11, 21]. This preparation is commonly used in our laboratory for studying live cells in a 3D experimental model mimicking an *in vivo* matrix microenvironment. To prepare 1 ml of neutralized collagen gel solution, 500 μl of type I collagen solution at 3 mg/ml were mixed with 100 μl of culture medium (10X concentration), 100 μl of NaHCO3 (22 g/l), 90 μl of 0.1 M NaOH, 10 μl of L-Glutamine (100X concentration), 100 μl of fetal calf serum, and 100 μl of H_2_O. A mixed solution, at a final concentration of 1.5 mg/ml type I collagen, was deposited into culture dishes and incubated during 30 min at 37°C to promote gel polymerization. For DRS experiments, 3D constructs were prepared with the same protocol except that the concentration of type I collagen solution was 5 mg/ml, giving a final gel concentration of 2.5 mg/ml.

### Kinetics of gel formation

The effect of collagen aging on the gelation process was studied by an *in vitro* fibrillogenesis assay that allowed a real-time monitoring of collagen polymerization after pH neutralization [27]. For that, different-age collagens were solubilised at 1 mg/ml in 0.018 M acetic acid (v/v) and 0.5 ml of collagen solution was mixed with an equal volume of 0.15 M phosphate buffer (pH 7.4) for each age condition. The mixed solutions were immediately transferred to the cell compartment of the Beckman DU640B spectrophotometer (Beckman, France). The system was maintained at 30°C by a heated water circulation to allow an optimal fibrillogenesis process. The kinetics of gel formation was monitored by recording absorbance at 400 nm every minute during 90 min. Turbidity was obtained by multiplying raw absorbance data by a factor of 2.303 [27].

### Spatially-resolved diffuse reflectance spectroscopy (DRS)

To assess the absorption and reduced scattering coefficients μ_a_ and μ_s_’ of biological samples at various wavelengths, an optical system based on a fibers bundle has been developed [24]. The easily portable system consists of a tungsten halogen lamp as excitation source (HL2000, Ocean Optics), a custom remote fiber optic probe for sample illumination and backscattered light collection, a fibered spectrometer for acquisition of the reflectance spectra (QE65000, Ocean Optics), and a computer for controlling measurement in less than five seconds and for data processing. The remote probe placed over the sample area under examination includes a central illumination fiber and a circular network of optical fibers located at various distances from the illumination fiber in order to collect light scattered from the 3D collagen construct. The reflectance signal at a specific wavelength follows an exponential decay as function of the distance between the detection and illumination fibers [36]. By comparison of this decay with the values given by a Look-Up Table previously constructed using Monte Carlo simulations, optical characteristics of observed specimens are evaluated. To apply the method to collagen hydrogels, we choose to assess their reduced scattering coefficient with an *a priori* absorption spectra close to zero [37]. Different-age collagen 3D matrices were prepared in polystyrene 24-well plates (1.5 ml of gel per well at 2.5 mg/ml for a compromise between ease of use and measurement sensitivity) as described above, then the probe was placed over each well for DRS measurement. Reflectance spectra were acquired at 3 different source-detector distances ranging from 1100 to 2000 μm.

### Confocal reflectance microscopy (CRM)

For each collagen-age, 3D matrices were prepared, directly in 2-well chambered 0.17 mm coverglass Lab-TeK systems (Nunc, France) according to 500 μl of gel/chamber at 1.5 mg/ml. Confocal reflectance microscopy was used to image samples after excitation at 488 nm (63X Plan-Apochromat oil objective N.A. 1.4) using a confocal laser scanning microscope Zeiss LSM 710 LNO (Carl Zeiss, Germany), driven by the ZEN Imaging Software (Carl Zeiss). The beamsplitter was set to NT 80/20 to switch in confocal reflectance mode. Images presented are the Maximum Intensity Projection on a 20 μm stack with z = 0.5 μm. Processing of confocal images and quantitative analysis of collagen networks were performed using ImageJ^®^ software.

### FTIR imaging in ATR mode

Different-age collagen 3D constructs were analyzed with the IR imaging system (Spotlight 300, Perkin Elmer) coupled to an ATR imaging device. The ATR set up consists of a Germanium crystal (600 μm diameter) with a high refractive index (*n* = 4.0) for the internal reflection of the IR beam. The crystal was put into contact with the sample, and spectra were recorded between 4000-750 cm^−1^ at a spatial resolution of 1.56 × 1.56 μm² nominal pixel size. For each pixel, 32 scans were averaged at 2 cm^−1^ resolution. Raw IR data were pre-processed as follows. An atmospheric correction was applied in order to remove contribution from water and CO_2_. Then spectra were baseline-corrected, offset corrected, and vector-normalized to be compared.
